# Validation of the Spanish Version of the Questionnaire on Environmental Awareness in Nursing (NEAT)

**DOI:** 10.3390/healthcare10081420

**Published:** 2022-07-29

**Authors:** Olga María Luque-Alcaraz, Antonio Gomera, África Ruíz, Pilar Aparicio-Martinez, Manuel Vaquero-Abellan

**Affiliations:** 1 Neurosurgery Department, University Hospital Reina Sofia’s, Andalusian Health Care System, 14004 Cordoba, Spain; 2 Service of Environmental Protection, Environmental Protection Office (SEPA), University of Córdoba, 14071 Cordoba, Spain; agomera@uco.es (A.G.); en1vaabm@uco.es (M.V.-A.); 3 IMIBIC GC 12 Research Groups of Clinical-Epidemiological Research in Primary Care, Biomedical Program for Occupational Medicine, Occupational Epidemiology and Sustainability, Nursing, Pharmacology, and Physiotherapy, Faculty of Medicine and Nursing of Cordoba, 14071 Cordoba, Spain; 4 Quantitative Methods for Economics and Business, Department of Applied Economics I. Sevilla University, 41004 Seville, Spain; africaruiz@us.es

**Keywords:** awareness, nursing, environmental health, climate change, sustainable development goals

## Abstract

Environmental awareness of the ecological problems caused by this climate crisis and its impact on global health has been growing globally. Nurses are health care agents that usually hurt the environment and contribute to the unsustainability of the care system. Such behavior is perpetuated without the nurses’ awareness and is even magnified by the current pandemic, jeopardizing the health systems and the Sustainable Development Goals. However, there is no Spanish version of any survey that measures the awareness of these agents, only the Nurses Environmental Awareness Tool (NEAT) is available. The current research presents a unique investigation based on a mixed method, using the Spanish version of the NEAT, also called NEAT-es. The results of the mixed analysis (N = 376), a cognitive interview, and descriptive analysis indicated perfect consistency (Cronbach’s alpha > 0.80), better than the original. The survey validation achieved higher values and can be used to measure environmental awareness in Spain and Spanish-speaking countries.

## 1. Introduction

The health sector significantly impacts the environment, generating a considerable climate footprint and directly impacting the population’s health. The global climate footprint from the health care sector represents more than 4.4% of net greenhouse gas (GHG) emissions since it is a great consumer of resources and energy [1].

In addition, the health sector is an excellent waste generator, and chemical products, such as single-use non-biodegradable plastic containers, increase the generation of microplastics [2,3]. Proper environmental awareness is the primary reason to avoid unsustainable health systems [4,5,6].

### Theoretical Foundation: Nursing, Awareness, and Sustainability

Since the beginning of modern nursing, environmental awareness has become a relevant issue to address in nursing. Florence Nightingale listed five critical elements for an environment to be considered healthy: fresh air, sunlight, clean water, waste disposal, and hygiene, and indicated that the environment is an essential factor to help recover or maintain good health [7,8,9]. This idea continues and has grown, as indicated by one of the statements of the International Code of Nurses, “Nurses contribute to the population’s health and work to achieve the Sustainable Development Goals” [10].

However, previous studies have shown how the health society, nurses, and other health professionals live in a paradox since they are both relevant agents in health and creators of significant amounts of waste and pollutants [8,11,12]. This paradox is known as environmental hyperopia among nurses [13,14]. Nurses take care of patients, but they do not seem to care so deeply about the environment surrounding them, even though all care has an intrinsic ecological impact [6,13,14]. Moreover, in case of further system pressure and life-threatening situations, such as the current pandemic, nurses continue to have a lack or little increase of environmental awareness. Additionally, nurses oversee administering and managing care as the health agents within the multidisciplinary team. It can significantly impact their patients’ environment and health if they are unaware. At the same time, if they maintain sustainable behavior, they can reduce their climate footprint and environmental problems to achieve the Sustainable Development Goals (SDGs) set for the agency for the year 2030 [15,16,17]. Recent articles have indicated that high waste production continues [6,13,14,18,19]. It could explain such difficulties via the disequilibrium between environmental sustainability and sanitary activity and the possibility of measuring nursing perception regarding environmental awareness in any country and moment [20].

This environmental awareness can be defined as the system of experiences and knowledge that individuals actively apply to their relationship with the environment [21]. It is a multidimensional concept that embraces all associated understanding, beliefs, values, attitudes, and behavior related to the environment. Therefore, it is a primary stimulus in searching for ways to attain sustainability, the measurement of this awareness among nurses being highly important. As indicated by the International Council of Nurses [22], nursing as a profession is committed to reducing its role in GHG and, therefore, its environmental awareness [23]. In this sense, environmental awareness is increasing the responsibility [24] to achieve a balance (ecological sustainability) in the healthy environment around us [15,25,26], to ensure the well-being of current and future generations [8].

Despite the previous contradictions, nursing throughout this decade has been aligning with the SDGs and raising awareness and taking responsibility for environmental problems [27,28]. However, it is more essential than before to measure the environmental awareness of nurses to provide sustainable improvements in their daily care practice, as has been reflected by the waste and consumption during the COVID-19 pandemic [13]. In this sense, this need has been highlighted during the pandemic and the high waste production [27,28], highlighting the reasoning and importance behind creating the questionnaire focused on nurses’ environmental awareness [29].

However, there is a reduced number of validated surveys focused on nurses’ perceptions regarding environmental impact, climate change, and awareness. The available surveys have been created in the United States of America, and there are not available in different languages [30,31]. Precisely, among the available validated questionnaires, the Climate, Health, and Nursing Tool, or CHANT [30,32], measures nurses’ perception of climate change and does not specifically evaluate environmental awareness. The same authors created other validated surveys measuring nurses’ perceptions and behavior related to environmental awareness. This specific survey focused on environmental awareness in nursing is called Nurse’s Environmental Awareness Tool (NEAT) questionnaire, which has three sub-scales (Nurse Awareness Scales: NAS; Nurse Professional Ecological Behaviors Scales: NPEB; Personal Ecological Behaviors Scales: PEB). The NEAT provides the necessary information to determine nurses’ environmental awareness [31], which is key to adequate protocols and activities to reduce the GHG produced by the health system and waste from nursing activities [15,16,17]. The NEAT was created and validated in the United States to measure environmental awareness in nursing, but only for English speakers, leaving out Hispanic populations. In this sense, other studies authors have indicated the relevance of having translated validated surveys for the work of nurses [29,33].

Based on the need to measure nurses’ environmental awareness and the lack of translated NEAT in other languages, the process for its validation in other languages is essential for several countries worldwide. Therefore, it is necessary to have a Spanish version of the NEAT that measures the environmental awareness of Spanish speakers since it is in the top four of the most spoken languages in the world [34]. Therefore, this study aims to develop and validate a Spanish version of the original English NEAT, guaranteeing conceptual, semantic, and contextual equivalence between both questionnaires. Additionally, the hypothesis, based on the consistency and validity of another survey, was that a validated Spanish version of the NEAT would be obtained.

## 2. Materials and Methods

### 2.1. Study Design

The research design was a qualitative and quantitative mixed method carried out. The qualitative part was carried out using cognitive interview (CI) techniques [35] to validate the content of the construct. In these semi-structured CIs, the participants were measured to ask about the level of understanding, completion, and presentation of the online format. Subsequently, the pre-test, which was included in the NEAT-es questionnaire of the pilot study, was intended to measure the face validity [36]. Three sections were included: a question that asked about the degree of difficulty in completing the questionnaire with a five-point Likert scale, where one = very difficult and five = very easy, one about the time to complete it; and an open question was included for participants to have comments in a text box, which focused on “Would you like to comment any further on the survey?”

**Figure 1:**
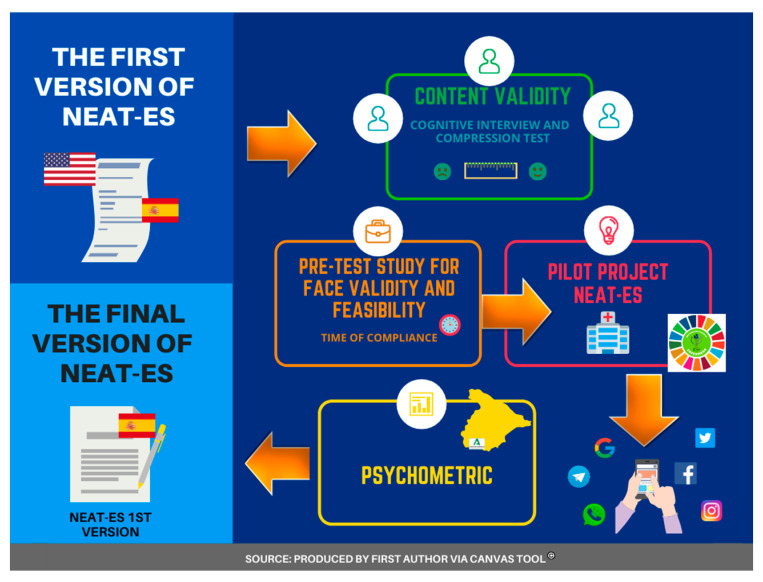
Study design and procedure followed for the validation.

Finally, a quantitative observational descriptive study was started to validate psychometry (reliability and factor analysis). The psychometric analysis was used, using the same criteria used by the original author [31,37], whose reliability was found through Cronbach’s alpha analysis and factor analysis using the maximum likelihood method [38], assuming that these factors are correlated, oblique rotation (Promax) was used [39]. This same psychometric methodology was carried out in the preliminary pilot project, the pilot study, and the samples by validation for the study exploratory factor analysis (EFA) and the confirmatory factor analysis (CFA) (Figure 1).

### 2.2. Sample

All the nursing staff, from nursing assistant technicians, nursing registered nurses, and students of both categories working or studying in health systems, mainly public hospitals, from Andalusia were approached to participate. Nurses were included in the study regardless of their contractual relationship with the hospital (contract or permanent staff) or training status, such as postgraduate nurses or specialists. The study excluded nurses whose primary work was not related to nursing competencies.

The sampling was based on the population of nurses in Spain in 2019, estimated at 388,153 nurses. From this population, the sampling was carried out using the GRANMO Sample Size Calculator (Program of Research in Inflammatory and Cardiovascular Disorders, Institut Municipal d’Investigació Mèdica, Barcelona, Spain) and Epidat version 4.2 (Servicio de Epidemiología de la Dirección Xeral de Saúde Pública da Consellería de Sanidade, Xunta de Galicia, Spain) [40], with a confidence level of 95% and a margin of error of 5.05%. From this, we found that we needed 376 nurses. Therefore, a representative, random and homogeneous sample of the Spanish nursing population was carried out. Intentional sampling was conducted, with data collection from November 2019 to March 2021.

To evaluate the test–retest reliability, 40 nurses were randomly selected for the initial evaluation of the translated version in the pre-test phase. After the pre-test phase, 63 nurses evaluated the first version of the survey, randomly selected from 376 nurses. For the construct validity, the total sample was used for the final evaluation of the survey.

### 2.3. Validation Process

The validation process followed the DETAC protocol [41] and the recommendations of Sousa, V. D. and Rojjanasrirat, W. [42]. In this sense, the translation, adaptation, and validation process followed the methodology of Lauffer et al. (2013) [40] to prevent bias during the validation process.

#### 2.3.1. Translation and Back Translation

In line with the methodological guidelines, two types of translation were implemented: direct, literal translation, and reverse translation. The direct translation was carried out by two bilingual translators who were experts in English to Spanish translations. The questionnaire was sent in an independent text, including sections to write the literal translation together with instructions about the aims of the study. After the translation into Spanish, the surveys were back translated to English and later evaluated by the experts. In this sense, two new translators, blind to the original questionnaire, one an English native from the United States of America and the other a nurse with a fluent command of Spanish, were separately sent the consensus version produced by the first translators. No contact was allowed between them, and they were unaware of the study’s purpose and the original questionnaire in English. This synthesis was then sent to the author of the original questionnaire.

#### 2.3.2. Participants of Cognitive Interview (CI)

The cognitive interview (CI) was done in a neutral room and at a table to get a fluid dialogue between the participants and the interviewer.

A multidisciplinary technical group of three environmental technicians and a nurse with expertise in sustainability or environmental education participated in the CI to validate the content of the survey after the translation and back translation version of the NEAT questionnaire. Environmental technicians mainly formed the multidisciplinary team since they formed the technical team for the verification.

This research team conducted the cross-analysis and discussion of the translation concerning the original version to check the reliability of the translation (direct and back translation). The process focused on the formulation of questions, on the one hand, using a 5-point Likert scale on item difficulty, scale of understanding the questionnaire, and difficulty of the test, and on the other, through open questions with cognitive interviews, as quality control. The multidisciplinary team recorded their opinions on the online form’s understanding, completion, and presentation, obtaining its first version by consensus. In the back translation, no differences were found from the original. 

However, the semantic, linguistic, concept, content, experimental, and cultural equivalence were analyzed by the research team formed by the panel of multidisciplinary experts. This team observed the need to adapt items A1 to A6 to the Spanish environment and context, as they contained data exclusively related to the United States of America. After extensive research, checking the existing literature, and consulting environmental experts, the items were contextualized for the Spanish territory regarding health, social health services, and others. The first consensus version of the questionnaire was obtained and denominated NEAT-es.v.1.

#### 2.3.3. Participants in End-Users Group Analysis and Pre-Test

With this first version, the end-users group analysis was conducted on five participants of the total pilot sample formed by nursing personnel with university degrees (registered or graduate nurses), nursing care technicians (nursing assistants), and students who perform practical work in both categories (student nursing specialists). The characteristics of the sample were primarily women with more than 20 years of experience and nurses with a university degree who worked in a public hospital in the morning shift, and they were chosen at random before sending the questionnaires to the final total sample to measure the degree of difficulty and the time to complete the questionnaire, using a Likert scale. This focus group evaluated the NEAT-es.v.1., showing a consensus about the usability of the survey.

After the end-users group analysis, the sample was increased and a pre-test was carried out. This analysis was performed with 40 participants, 52.5% women and 47.5% men, with an average age of 44.53 ± 1.9. Only 27.5% had less than ten years of working experience. Additionally, 87% were nurses, 7.5% were nursing students, 5% were nursing care technicians, 62.5% worked in the hospital, 22.5% in primary care, and 15% in others. Although this study was not conclusive regarding the validation process, it was consistent. After this inconclusive data, the sample was expanded to a total of 63 participants, that is, a more significant number of the questionnaire items to obtain validity, carrying out a pilot analysis with this sample.

#### 2.3.4. Participants of the Pilot Study and Final Sample

The sample for the pilot study consisted of 63 nurses (registered or graduate nurses), nursing care technicians (nursing assistants), students who perform practical work in both categories, and students as nursing specialists. All other categories were excluded. This pilot sample comprised 38.10% men and 61.90% women, most of whom were between 30 and 40 years old, and 40.03% had more than 20 years of work. In addition, 76.19% belonged to the capital’s public health or surroundings (Table 1). With this sample (n = 63), the face validity was measured, and a Likert scale was performed on the difficulty of the test. 

The final sample consisted of 376 participants with the same personnel characteristics as the pilot study. From this sample of 376, one-third was randomly taken for the export validation study. The exploratory factor analysis (EFA), based on two-thirds of the total participants, were selected patients (n = 251) and confirmatory factor analysis (CFA) with the selection of final participants (N = 376). The majority were women with more than 20 years of experience, nurses with a university degree, who worked in a public hospital, and whose sociodemographic characteristics are described in Table 1.

The sociodemographic data of both samples indicated homogeneity among the nurses and similar data regarding the years of experience and working in public centers. Such sociodemographic data are relevant factors that contribute to the validation of a survey, especially in environmental awareness [43], being similar to the NEAT validation process [31] and relevant since the term was introduced two decades ago [44].

### 2.4. Nurse’s Environmental Awareness Tool

First, to determine the current degree of ecological awareness among nurses, it was necessary to identify an adequate survey. Therefore, diverse databases (PubMed, Web of Science, Scopus, and others) were reviewed, and we identified only one survey in the BiblioPRO library (Biblio-Pro, 2021). However, we did not find a Spanish version of such a questionnaire. The validated questionnaire in English found to measure the environmental awareness of nursing personnel is called the Nurses Environmental Awareness Tool (NEAT) [31,37]. The NEAT questionnaire allows the measurement and evaluation of environmental awareness in nursing, as it consists of a series of ecological awareness scales specifically developed for nurses. The NEAT questionnaire is self-administered and is divided into three scales: “Nurse Awareness Scales” (NAS), “Nurse Professional Ecological Behaviors Scales” (NPEB), and “Personal Ecological Behaviors Scales” (PEB).

First, the (NAS) focuses on determining nurse awareness and it consists of 11 items. The items focus on statements related to two questions (“Have you heard of this information before?” and “How related to health impacts do you think this is?”), being answered on a five-point Likert scale, with one = never/not at all and five = definitely/a lot.

The second, the NPEB scale, measures the professional behavior of nurses to mitigate environmental effects and consists of nine items, presented as affirmations with two questions, (“How often do you do this behavior?” and “How easy or difficult is this behavior to do?”), being also answered using a five-point Likert scale. The third and final scale, the PEB based on ecological behavior, with 11 items and the same questions as the NPEB.

Permission was granted by the author of the original NEAT questionnaire to translate it into Spanish, now denominated as NEAT-es in all its different versions. For the validation, the NEAT-es questionnaire was distributed online through a subscription-based platform (Google), available via a link, and accessible by the participants in Spain. Additionally, the quick response (QR) code was created based on the link and located in hospitals and other centers across the country in person and online through direct messages via social media (such as Facebook, Twitter, or Instagram).

### 2.5. Data Analysis

#### 2.5.1. Qualitative Study: Cognitive Interview

The cognitive interviews of the multidisciplinary group were collected in a field notebook and recorded. This interview contained a question about the five-point Likert scale, where one = very difficult and five = very easy, to verify the degree of difficulty and understanding of the items in the final questionnaire and an open question. This open question followed a transcription process using the ATLAS.ti version 9 software. The Microsoft Word 2019 software (Microsoft CLUF (EULA), Albuquerque, NM, United States) was used for the Likert scale. Then the prioritization process was followed to produce a single final version for each item. After the cognitive interviews, a triangulation process was carried out between techniques and researchers to add objectivity and validity to our research.

#### 2.5.2. Pre-Test Study and Pilot Study

The pretest was included in the NEAT-es questionnaire of the pilot study. It was intended to measure the face validity in which three sections were included: a question with a five-point Likert scale, which asked for the degree of difficulty when completing the questionnaire, one on the time to complete it, and finally an open question was included for the participants to have comments in a text box.

#### 2.5.3. Statistical Analysis: Descriptive and Psychometrics for Final Validation

Several methods were used for the final validation: reliability (internal consistency) was verified by Cronbach’s alpha and was followed by two factorial analyzes that evaluated the factorial structure of 62 items (31 items with two responses each). On the one hand, an exploratory factor analysis (EFA) with 2/3 of the total sample of participants was selected, that is, a sample of n = 251 patients, and on the other a confirmatory factor analysis (CFA) with the sample of n = 376 participants. For the validity of the construct for the EFA and CFA was carried out using the Statistical Package for Social Science (SPSS) (IMB, Endicott, Nueva York, United States of America) for the CFA and R commander, using the R package [45], via the lavaan package (V.3.5.0), for the CFA with the same method used by the author of the original NEAT; that is, the maximum likelihood method was used for the extraction of the factors present an oblique rotation (Promax) was used [31,37]. Moreover, other statistical analyses used for the CFA of the validation were implemented such as the chi-square goodness of fit statistics, comparative fit index (CFI), goodness of fit index (GFI), Tucker–Lewis index (TLI), root mean squared error of approximation (RMSEA), and its respective *p*-value or the root mean square residuals (RMSR). Finally, convergent and discriminant validity were evaluated via the average variance extracted (AVE) and heterotrait monotrait ratio (HTMT). For such analyses, the R studio, PROGRAMA, and Programa2Salida were implemented by the researchers [45].

### 2.6. Ethical Considerations

The research will respect the principles of Bioethics of the Oviedo Convention, the Helsinki Declaration, and the current Spanish Data Protection Laws (5 December 2018). The participant’s confidentiality is always acknowledged, and their data are dealt with anonymously. The study was approved by the Reginal Biomedical Research Ethics Coordinating Committee (No. 267, ref. 3605). Additionally, it is part of the doctoral thesis project called “The Nursing Responsibility in the Environmental Sustainability” of the Biomedicine doctoral program.

## 3. Results

### 3.1. Qualitative Study: Cognitive Interview

After asking the multidisciplinary team in the cognitive interviews about the level of understanding, completion, and presentation of the online format, four “easy” answers were obtained on the five-point Likert scale, where one = very difficult and five = very easy. A similar result was obtained for the open question, with only the comments about the excessive number of items, 62 in total (31 answers, with two solutions each). The mean time to complete the NEAT-es questionnaire was 8.7 (±1.9) minutes.

### 3.2. Pre-Test and Pilot Study

After obtaining the NEAT-es first version questionnaire, it was tested in a pre-test by five nursing professionals, who estimated their level of understanding and the suitability of the format (face validity), obtaining a score of 4 (easy) for each of the questions in the five-point Likert scale, indicating a good level of understanding when completing the questionnaire. In this case, the mean time to complete the NEAT-es questionnaire was 9.6 (±2.7) minutes, the same as the expert results and the original NEAT. In addition, the degree of difficulty of the NEAT-es-v.1 (the first version) was established. The questionnaire was included in the pilot of 63 participants. A score of 3.8 was obtained in “comprehension” on the five-point Likert scale, where one = very difficult and five = very easy.

A preliminary exploratory pilot study with n = 40 participants was carried out. Despite having high consistency (Cronbach’s alpha = 0.909), we did not obtain good results in the factorial analysis (0.455–0.597), perhaps due to the high number of elements (62 items) and the small sample size (n = 40). When the sample was expanded to 63 participants, better internally consistent results were obtained (Cronbach’s alpha) for each sub-scale with two questions: NAS-es = 0.832/0.889; NEPB-es = 0.805/0.703; PEB = 0.809/0.738. The factory analysis with results between 0.013 and 0.980, so it was decided to continue expanding the sample size due to the high consistency of the questionnaire.

### 3.3. Result Psychometric for Final Validation: Reliability and Exploratory Factor Analysis (EFA) and Confirmatory Factor Analysis (CFA)

Cronbach’s alpha checked reliability (internal consistency) and a factor analysis assessed the factor structure of the 63 items. For reliability (internal consistency) of NEAT-es v.1. and the 62 items, Cronbach’s alpha was estimated and the internal consistency was internally consistent (Cronbach’s alpha for each sub-scale > 0.80). The questionnaire could follow a similar behavior regarding its metric equivalences to the original [31,37].

An exploratory study of EFA was carried out with n = 251 participants, and CFA with = 376 participants, for each of the three sub-scales, NAS-es, NPEB-es, and PEB-es with two questions for each item are collected in the following sections with their corresponding table.

#### 3.3.1. NAS-es Scale: Reliability, EFA, and CFA

The internal consistency estimated by Cronbach’s alpha for the two NAS-es questions was high in both EFA/CFA factorial analyses. When asked for awareness, it was 0.886/0.891, and when asked for health, it was 0.891/0.886. Both factor analyses, EFA and CFA of the NAS-es, show high significance since a *p*-value of 0.000 or a lower *p*-value is obtained, making it significant; in addition, there are no items below 0.4 or 0.3, as recommended by the author of the NEAT questionnaire, so the saturation of the items is adequate (Table 2 and Table 3).

**Figure 2:**
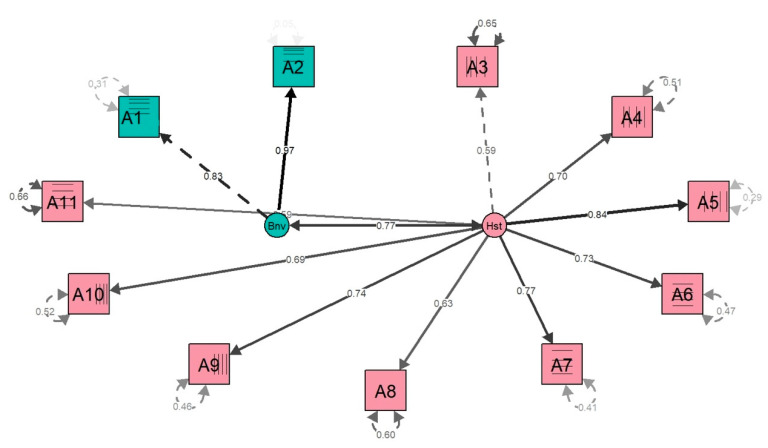
Factors graph of the NEAT-es NAS for the CFA. Note: A1, A2, A3, A4, A5, A6, A7, A8, A9, A10 and A11 correspond to the factors of the NAS-es scale of the NEAT-es.

The EFA indicated values on the limit in 1 point in the second pattern or factor in A1, so we analyzed the discrepancy in the CFA (Table 3), for which not only Tukey but other analyses were implemented. The RMSEA indicated great values, accepting the model with the adequation of the factors with values lower than 0.3. The chi-square fitness was 957.064 (*p* < 0.001), with a good TLI (0.973), AIC (935.565), BIC (9364.431), and SRMR (0.031) for awareness. For “Health”, the two-factor model of the items indicated acceptable values since the chi-square fitness was 1025.294 (*p* < 0.001), with a good TLI (0.964), AIC (5481.647), BIC (5540.591), and SRMR (0.041). The factors graph represents the two factors obtained in the study, indicating the mode of the number of factors that must be chosen (Figure 2).

Moreover, the reliability and validity were good for both sub-scales (AVE = 0.69; HTMT = 0.763; heterotrait correlation = 0.478). 

#### 3.3.2. NPEB-es Scale: Reliability and Exploratory and Confirmatory Factor Analysis

The internal consistency of the NPEB-es questionnaires was estimated using Cronbach’s alpha. For each factor analyzed, AFE and CFA show high significance (*p*-value < 0.001), which makes it significant; in addition, even though values below 0.4 or 0.3 were found as recommended by the author, it was decided not to extract any factor to adapt it to the original, since the US version was already validated. From Table 4 and Table 5, the consistency of each factor was determined, indicating an excellent and good internal consistency. Despite the difference between factors, Cronbach’s alpha was higher than the range considered acceptable, being good and in some cases being close to excellent. Additionally, the results of sub-scale behavior and difficulty (Table 5) indicated an acceptable model for the items (chi-square fitness was 347.440 (*p* < 0.001), with a good TLI (0.973), AIC (6728.602), BIC (6779.582), and SRMR (0.039) for sub-scale behavior vs. chi-square fitness 563.581 (*p* < 0.001), TLI (0.93), AIC (9278.305), BIC (9344.972), and SRMR (0.044) for difficulty). The reliability and validity were good for both sub-scales (HTMT = 0.636 and heterotrait correlation = 0.278)

#### 3.3.3. PEB-es Scale: Reliability and Exploratory and Confirmatory Factor Analysis

Table 6 and Table 7 focus on the reliability and confirmatory factor analysis of each factor for the Nurse Professional Ecological Behavior (PEB) and the Nurse Professional Behavior (PEB) difficulty scales EFA and the relatedness to health scale for 251 (Table 6) and the final sample of 376 participants (Table 7). Both tables showed how the items of each sub-scale (behavior and difficulty) were acceptable (over 0.5). Only in the case of C11 for the structure of the difficulty, was the obtained value low in both cases. Only in some cases, such as factor C11 in the behavior section (value = 0.029) (Table 7), do the data indicate lower relevance when compared to other factors, such as factor C3 (value = 0.690). Moreover, the results of the Barlett’s sphericity test showed a high significance (*p*-value < 0.001), and the Cronbach’s alphas were good for the sub-scale behavior (value = 0.831 in Table 6 and value = 0.825 in Table 7) and acceptable for the difficulty (value = 0.783 in Table 6 and value = 0.774 in Table 7). Additionally, the sub-scale behavior indicated regarding RMSEA is acceptable (0.066), with the *p*-value of the RMSEA adequate (0.06), matching acceptable values of chi-square fitness (1012.978, *p*< 0.001), TLI (0.925), AIC (11011.744), BIC (11094.266), and SRMR (0.043), being similar but lower for difficulty (RMSEA = 0.066, with *p*-value of RMSEA = 0.055). Finally, the reliability and validity were acceptable for both sub-scales (HTMT = 0.676 and heterotrait correlation = 0.321)

The final version of the questionnaire in Spanish was obtained, known as “NEAT-es”, or in its final version “NEAT-es.v.1.” (Appendix A), based on the results of the final sample (Table 7) and the significance of the results presented through the tests (*p*-value < 0.001; Cronbach’s alphas > 0.7), the low values of some items studied in the final analysis were not insignificant.

## 4. Discussion

The current research has presented the Spanish validation of the NEAT through psychometric validation in Spanish, which has been described as a key tool for determining environmental awareness among nurses and therefore having a future positive effect on environmentally sustainable systems.

The results indicated that all the items were rated above good ranking, making it an excellent tool to measure Spanish nurses’ awareness. Despite being unable to compare to other validated versions of the NEAT, these results are highly interesting since other validations have indicated that more than two items usually tend to have Cronbach’s alphas under 0.7 in the score of patterns [33,46], which suggests that this validation provides a high-quality translated survey.

The preliminary descriptive stage indicated that internal consistency was also good, with a higher Cronbach’s alpha (0.90) and the subsection was more relevant. However, the EFA of this preliminary analysis, being standardized that the factor loadings are between -1 and 1, identified values on the limit of 1 point, usually in pattern 2. These results can be explained by the obliquely rotated factorial solution, which indicated the association of two latent factors (patterns 1 and 2) that group all the variables, surpassing the factorial loads [47]. Despite the initial surpassing of the factorial loads, the confirmatory analysis showed factor loads between the standardized limits, confirmed in complementary studies that were represented only once (Figure 2), but also confirmed in all the analyses with the R commander.

Moreover, the consistency and validity of the NEAT-es have indicated similar results to the creators of the NEAT [30,32]. Although no previous study has validated NEAT in other languages, the creation and validation of the original NEAT indicated a high consistency in matching the current findings regarding the sub-scales [31,37]. The similarities between the original NEAT and NEAT-es could be interpreted as the result of a satisfactory validation of the Spanish version, in sync with the initial hypothesis.

These results are relevant since the previous analysis of English-speaking nurses indicated that they are conscious of their significant impact on their daily work and have skills to mitigate them [30,32] and their insufficient knowledge about the questionnaire topics. Nonetheless, such results could not be compared since the NEAT is unavailable in other languages. Still, the findings indicated that the NEAT-es questionnaire is a suitable tool to measure and correct environmental deficits in the daily care of nurses.

Additionally, the psychometrics data related to ecological awareness have highlighted the overlooked nurses’ experiences regarding their competencies, mainly skills, knowledge, and aptitudes. Despite having present knowledge, the skills and application of such knowledge require further investment and application in actual conditions. These findings could be associated with the spread and pressure suffered during the pandemic, which contributes to the worsening of the health care systems and the health of the professionals [48]. Such a situation causes difficulty in carrying out sustainable procedures, worsening the nurses’ sustainable awareness [49,50]. Therefore, environmental awareness and sustainability through the SDGs for nurses, having under consideration the multidisciplinary concept in health care systems [21], could be the most effective measure for community engagement and modification of unsustainable behaviors [24,37].

As with any research, the current study presents limitations. The limitation of the research is the approach taken for the validation of the survey. The methodology of pre-data collection strategy, including CI to analyze an instrument, is relatively recent, requiring additional reproduction with other instruments. The survey validation occurred partially during the beginning of the pandemic, with the instrument’s validity linked to the cross-cultural approach.

Despite these limitations, the current NEAT-es has implications for the SDGs, policies, and nursing education via understanding the current view of nurses. Moreover, since there is much-needed improvement in education, environment, and nursing training [51], this survey can promote more research on environmental sustainability in health care [52,53,54]. It refers to the fact that it is a Spanish version adapted in the Spanish territory; however, it is possible that it can be used for other Spanish-speaking countries as translated.

## 5. Conclusions

A Spanish version of the NEAT questionnaire was obtained, which was the objective of this research, and was renamed the NEAT-es questionnaire, which has been validated using psychometric characteristics. This questionnaire could help measure Spanish nurses’ environmental awareness and contribute to health teams’ environmental awareness. The NEAT-es questionnaire was tested in a pilot project with a high completion rate and good compression results, obtaining the final version of the NEAT-es questionnaire with a four-point Likert scale (accessible). The Likert scale referred to the difficulties in the questionnaire and was distributed on the Google Forms platform.

The first version of NEAT-es has been developed and psychometrically tested and is ready for further use and study in Spanish or Spanish-speaking populations. There is no questionnaire to measure environmental awareness in nursing specifically in Spain, so it is interesting to obtain it to measure environmental awareness in Spain. Additionally, the questionnaire can be adapted to Spanish-speaking countries.

## Figures and Tables

**Table 1:** Sociodemographic characteristics of the pilot project and final sample.

	Pilot Project (N = 63)	Final Sample (N = 376)
**Age**	40.76 (13.78)	37.7 (0.62)
**Gender**		
Female	39 (61.9%)	275 (73.1%)
Male	24 (38.1%)	101 (26.9%)
Non-binary	0	0
**Working experience (years in the field)**		
More than 20 years	29 (46.0%)	142 (37.8%)
Between 11 and 20 years	9 (14.3%)	65 (17.3%)
Between 10 and 5 years	3 (4.8%)	51 (13.5%)
Less than 5 years	22 (34.9%)	118 (31.4%)
**Occupation**		
Nursing Assistant	4 (6.3%)	23 (6.1%)
Nursing of Assistant Student	1 (1.6%)	1 (0.3%)
Nurse with Certificate from University	41 (65.1%)	267 (71.0%)
Nursing Student to Obtain University Certificate	15 (23.8%)	72 (19.1%)
Nursing Specialist Students	2 (3.2%)	13 (3.5%)
**Workplace**		
Local Hospital	28 (44.4%)	139 (37.0%)
Local Primary Health Care	10 (16.0%)	68 (18.1%)
Regional Hospital	14 (22.2%)	124 (33.0%)
Regional Primary Health Care	2 (3.2%)	12 (3.2%)
Socio-Sanitary (i.e., hospice)	5 (7.9%)	14 (3.7%)
Others	4 (6.3%)	19 (5.0%)
**Center Financial status**		
Public	48 (76.2%)	330 (87.9%)
Private	8 (12.7%)	20 (5.2%)
In Collaboration with Public and Private entities	7 (11.1%)	26 (6.9%)
**Work shift**		
Only Morning	30 (47.6%)	185 (49.2%)
Only Afternoons	4 (6.3%)	18 (4.8%)
Only Nights	2 (3.2%)	11 (2.9%)
Rotating Shift (switch between other shifts)	24 (38.1%)	160 (42.6%)
Others	3 (4.8%)	2 (0.5%)

**Table 2:** Factor loadings and Cronbach’s alphas for the Nurse Awareness Scale (NAS-es) of the questionary NEAT-es 1st version EFA (n = 251 participants).

NAS-es SCALE									
Validation EFA									
NAS-e		Awareness				Health			
Items		Loadings							
Factor		Pattern		Structure		Pattern		Structure	
		1	2	1	2	1	2	1	2
A1		−0.119	1.0	0.565	0.995	0.979	−0.176	0.869	0.431
A2		0.154	0.659	0.575	0.757	0.957	−0.128	0.877	0.466
A3		0.457	0.119	0.533	0.410	0.586	0.186	0.701	0.549
A4		0.614	0.008	0.619	0.400	0.368	0.396	0.614	0.625
A5		0.585	0.263	0.752	0.636	0.607	0.231	0.751	0.608
A6		0.263	0.149	0.653	0.505	0.351	0.386	0.590	0.603
A7		0.767	−0.002	0.766	0.487	0.210	0.537	0.543	0.667
A8		0.661	−0.010	0.655	0.412	0.028	0.602	0.401	0.619
A9		0.720	−0.071	0.675	0.389	0.008	0.688	0.436	0.693
A10		0.608	0.014	0.617	0.403	−0.036	0.832	0.480	0.809
A11		0.601	−0.024	0.585	0.359	−0.159	0.740	0.300	0.641
**Kaiser–Meyer–Olkin measure of sampling adequacy**		0.879				0.892			
**Bartlett’s Sphericity Test**	**Statistic**	1217.385				1404.342			
	***p*-value**	<0.001				<0.001			
**Cronbach’s Alpha**		0.886				0.891			

**Table 3:** Factor loadings and Cronbach’s alphas for the Nurse Awareness Scale (NAS-es) of the questionary NEAT-es 1st version CFA (n = 376 participants).

NAS-es SCALE									
Validation CFA									
NAS-es		Awareness				Health			
Items		Loadings							
Factor		Pattern		Structure		Pattern		Structure	
		1	2	1	2	1	2	1	2
A1		0.979	−0.176	0.869	0.431	0.944	−0.155	0.853	0.401
A2		0.957	−0.128	0.877	0.466	0.940	−0.131	0.862	0.422
A3		0.586	0.186	0.701	0.549	0.621	0.134	0.699	0.499
A4		0.368	0.396	0.614	0.625	0.460	0.272	0.621	0.543
A5		0.607	0.231	0.751	0.608	0.648	0.187	0.757	0.568
A6		0.351	0.386	0.590	0.603	0.291	0.419	0.538	0.590
A7		0.210	0.537	0.543	0.667	0.163	0.535	0.478	0.631
A8		0.028	0.602	0.401	0.619	−0.013	0.638	0.363	0.631
A9		0.008	0.688	0.436	0.693	0.042	0.681	0.443	0.706
A10		−0.036	0.832	0.480	0.809	−0.016	0.774	0.440	0.765
A11		−0.159	0.740	0.300	0.641	−0.141	0.743	0.296	0.660
**Comparative Fit Index (CFI)**		0.98				0.98			
**RMSEA**	**Statistic**	0.049				0.068			
	***p*-value**	0.48				0.123			
**Cronbach’s Alpha**		0.891				0.886			

**Table 4:** Factor loadings and Cronbach’s alphas for the Nurse Professional Ecological Behaviors Scales (NPEB-es) of the questionary NEAT-es 1st version EFA (n = 251 participants).

NPEB-es SCALE											
Validation EFA											
		Behavior				Difficulty					
Items		Loadings									
Factor		Pattern		Structure		Pattern			Structure		
		1	2	1	2	1	2	3	1	2	3
B1		0.113	0.408	0.338	0.471	0.109	0.445	0.052	0.297	0.502	0.258
B2		0.247	0.206	0.361	0.342	−0.045	10.0	−0.129	0.268	0.989	0.201
B3		−0.004	0.677	0.369	0.675	0.104	0.109	−0.234	0.022	0.068	−0.144
B4		−0.046	0.881	0.440	0.855	0.048	0.062	0.796	0.482	0.348	0.842
B5		0.552	0.226	0.677	0.530	0.449	0.027	0.258	0.592	0.276	0.499
B6		0.769	−0.075	0.728	0.349	0.722	−0.010	−0.200	0.615	0.184	0.169
B7		0.805	−0.004	0.803	0.439	0.837	−0.032	−0.088	0.780	0.241	0.333
B8		0.593	−0.007	0.589	0.320	0.665	0.060	−0.065	0.653	0.278	0.298
B9		0.350	0.055	0.380	0.248	0.458	0.023	0.146	0.542	0.238	0.390
**Kaiser–Meyer–Olkin measure of sampling adequacy**		0.826				0.774					
**Bartlett’s Sphericity Test**	**Statistic**	600.988				481.724					
	***p*-value**	<0.001				<0.001					
**Cronbach’s Alpha**		0.799				0.730					

**Table 5:** Factor loadings and Cronbach’s alphas for the Nurse Professional Ecological Behaviors Scales (NPEB-es) of the questionary NEAT-es 1st version CFA (n = 376 participants).

NPEB-es SCALE									
Validation CFA									
		Behavior				Difficulty			
Items		Loadings							
Factor		Pattern		Structure		Pattern		Structure	
		1	2	1	2	1	2	1	2
B1		0.131	0.350	0.300	0.413	0.074	0.580	0.328	0.612
B2		0.271	0.233	0.384	0.364	−0.009	0.776	0.331	0.772
B3		−0.085	0.639	0.224	0.598	−0.039	0.194	0.046	0.177
B4		0.021	0.809	0.412	0.820	0.370	0.194	0.455	0.356
B5		0.504	0.222	0.611	0.466	0.520	0.082	0.556	0.310
B6		0.797	−0.080	0.758	0.305	0.679	−0.067	0.649	0.231
B7		0.812	−0.054	0.785	0.338	0.830	−0.089	0.791	0.275
B8		0.594	0.006	0.597	0.293	0.682	−0.013	0.676	0.286
B9		0.388	0.059	0.417	0.246	0.484	0.051	0.507	0.264
**Comparative Fit Index (CFI)**		0.966				0.953			
**RMSEA**	**Statistic**	0.061				0.06			
	***p*-value**	0.259				0.219			
**Cronbach’s Alpha**		0.780				0.744			

**Table 6:** Factor loadings and Cronbach’s alphas for the Nurse Professional Ecological Behavior (PEB-es) of the questionary NEAT-es 1st version EFA (n = 251 participants).

PEB-es SCALE													
Validation EFA													
		Behavior						Difficulty					
Items		Loadings											
Factor		Pattern			Structure			Pattern			Structure		
		1	2	3	1	2	3	1	2	3	1	2	3
C1		−0.005	−0.037	1.0	0.413	0.364	0.999	0.077	0.122	0.487	0.351	0.272	0.547
C2		0.012	0.120	0.450	0.286	0.307	0.503	0.050	−0.037	0.879	0.400	0.184	0.892
C3		0.518	0.297	−0.007	0.709	0.633	0.337	0.702	−0.041	0.026	0.690	0.360	0.312
C4		0.368	0.430	0.021	0.658	0.679	0.352	0.708	−0.010	0.070	0.732	0.404	0.366
C5		0.274	0.090	0.050	0.355	0.289	0.205	0.321	0.081	0.019	0.375	0.266	0.172
C6		0.668	−0.001	−0.071	0.637	0.407	0.219	0.398	0.354	−0.079	0.565	0.561	0.167
C7		0.868	−0.125	0.052	0.809	0.463	0.380	−0.131	10.0	0.100	0.499	0.993	0.274
C8		0.767	−0.045	0.020	0.747	0.464	0.336	0.392	0.499	−0.118	0.623	0.694	0.156
C9		0.052	0.704	0.030	0.525	0.750	0.333	0.457	−0.030	0.108	0.485	0.251	0.293
C10		0.266	0.272	−0.050	0.423	0.427	0.174	0.311	0.118	0.008	0.381	0.295	0.165
C11		−0.129	0.823	0.029	0.421	0.751	0.301	0.079	0.067	0.019	0.125	0.115	0.067
**Kaiser–Meyer–Olkin measure of sampling adequacy**		0.861					0.811						
**Bartlett’s Sphericity Test**	**Statistic**	913.469					671.674						
	***p*-value**	<0.001					<0.001						
**Cronbach’s Alpha**		0.831					0.783						

**Table 7:** Factor loadings and Cronbach’s alphas for the Personal Ecological Behaviors Scales (PEB-es) of the questionary NEAT-es 1st version CFA (n = 376 participants).

PEB-es SCALE											
Validation CFA											
		Behavior				Difficulty					
Items		Loadings									
Factor		Pattern		Structure		Pattern			Structure		
		1	2	1	2	1	2	3	1	2	3
C1		0.191	0.324	0.405	0.450	0.021	−0.035	0.699	0.270	0.336	0.689
C2		0.115	0.273	0.295	0.349	−0.020	0.083	0.644	0.278	0.401	0.679
C3		0.363	0.401	0.628	0.641	−0.033	0.704	0.011	0.387	0.690	0.359
C4		0.194	0.587	0.581	0.714	0.025	0.718	0.020	0.457	0.743	0.398
C5		0.248	0.153	0.349	0.317	0.169	0.200	0.022	0.295	0.311	0.189
C6		0.550	0.102	0.617	0.465	0.367	0.361	−0.072	0.552	0.541	0.255
C7		0.878	−0.105	0.808	0.474	0.990	−0.148	0.068	0.928	0.471	0.374
C8		0.718	0.030	0.738	0.504	0.545	0.317	−0.081	0.701	0.597	0.292
C9		−0.013	0.752	0.483	0.744	0.000	0.374	0.236	0.312	0.495	0.428
C10		0.233	0.276	0.414	0.429	0.143	0.202	0.179	0.331	0.378	0.337
C11		−0.116	0.756	0.383	0.679	0.013	0.009	0.025	0.028	0.029	0.035
**Comparative Fit** **Index (CFI)**		0.943				0.94					
**RMSEA**	**Statistic**	0.066				0.07					
	***p*-value**	0.06				0.055					
**Cronbach’s Alpha**		0.825				0.774					

## Data Availability

The data presented in this study are available on request from the corresponding author. The data are not publicly available due to privacy restrictions.

## Appendix A

The final version of the questionnaire in Spanish NEAT-es.

**1.-NAS: Escala de conciencia del personal enfermero.**			
**Ítems**	**Por favor. lea las afirmaciones y conteste:** **Responda con la escala de la derecha.**	**¿Ha oído hablar antes de esta información?** **Escala likert:** **1.-No. nunca he oído hablar.** **5.-Sí. definitivamente he oído hablar.**	**¿Cómo cree que esto impacta sobre la salud?** **Escala likert:** **1.-Nunca** **5.-Mucho**
A1	De acuerdo con el Ministerio de Energía. la atención sanitaria se sitúa como el cuarto mayor consumidor de la energía dentro del sector servicios.		
A2	Los hospitales y centros de salud usan un 2% del consumo energético total. lo que supone un 30% respecto el sector de los edificios.		
A3	La mayor parte de la energía consumida en España. incluida la del sector sanitario. se basa en fuentes no renovables.		
A4	Cerca del 80% de los españoles se desplaza en vehículo privado (coche o moto). La energía utilizada puede igualar o exceder la energía requerida para el funcionamiento de un edificio de oficinas (incluyendo un hospital).		
A5	La energía utilizada en el transporte de productos médicos. alimentos y suministros representa una parte significativa de la energía total utilizada en la asistencia sanitaria.		
A6	Los hospitales españoles y centros de salud producen más de 700 toneladas de residuos al día.		
A7	Los productos químicos tóxicos usados en la asistencia sanitaria han contribuido a la acumulación de Mercurio. Dioxinas y Ftalatos en nuestro medio ambiente.		
A8	En las analíticas de sangre y orina. el personal enfermero puede mostrar niveles de algún agente químico tóxico.		
A9	Algunos plastificantes que ablandan los plásticos para facilitar su uso (por ejemplo en tubos para muestras sangre) son disruptores hormonales.		
A10	El Triclosán. una sustancia antibacteriana presente. por ejemplo jabones. está siendo objeto de estudio por su posible alteración hormonal.		
A11	La comida servida de manera convencional en hospitales puede contener restos de pesticidas y herbicidas.		
**2.-NPEB: Escala de comportamientos ecológicos profesional del personal enfermero.**			
Ítems	Por favor. lea las afirmaciones y conteste: Responda con la escala de la derecha.	¿Con qué frecuencia lo hace? Escala Likert: 1.-Nunca 5.-Siempre	¿Cómo de difícil o de fácil le resulta hacerlo? Escala Likert: 1.-Muy Difícil 5.-Siempre
B1	En el trabajo. apago las luces conscientemente cuando no están en uso.		
B2	En el trabajo. apago los monitores del ordenador cuando no están en uso.		
B3	En el trabajo. reciclo.		
B4	En el trabajo. motivo a mis compañeros/as para reciclar.		
B5	Trabajo para reducir el uso de los agentes químicos tóxicos en el hospital (tales como el Mercurio. DEHP o Triclosán).		
B6	Hago búsquedas en la literatura o en la web sobre agentes químicos tóxicos utilizados en la asistencia sanitaria.		
B7	En el trabajo. informo a otros miembros del personal sobre agente químicos tóxicos.		
B8	En el trabajo. educo a los/as pacientes sobre riesgos de exposiciones ambientales tales como los productos químicos tóxicos o la contaminación.		
B9	En el trabajo. animo al servicio de hostelería a servir alimentos locales.		
**3.-PEB: Escalas de comportamientos ecológicos personales**			
Ítems	Por favor. lea las afirmaciones y conteste: Responda con la escala de la derecha.	Con qué frecuencia lo hace? Escala likert: 1.-Nunca 5.-Siempre	¿Cómo de difícil o de fácil le resulta hacerlo? Escala likert: 1.-Muy Difícil 5.-Siempre
C1	En casa. calculo cuántos kWh de electricidad consumo.		
C2	Periódicamente. realizo el mantenimiento de mis tuberías para comprobar las fugas de agua y hago las reparaciones necesarias.		
C3	En casa. compro productos reciclados.		
C4	En casa. tomo decisiones en las compras teniendo en cuenta evitar la producción de residuos.		
C5	En casa. no uso pesticidas ni herbicidas.		
C6	En casa. compro productos ecológicos.		
C7	En casa. evito el uso de productos de cuidado personal que contengan productos químicos.		
C8	En casa. uso productos de limpieza respetuosos con el medioambiente.		
C9	Leo sobre temas relacionados con el medio ambiente y salud en los medios de comunicación.		
C10	Soy voluntario/a en acciones para apoyar un medio ambiente saludable (participo en Asociaciones. Organizaciones No Gubernamentales (ONG). etc).		
C11	Como enfermera/o. debato cuestiones sobre medio ambiente y salud con mis amistades y familiares.		
